# Viral metacommunities associated to bats and rodents at different spatial scales

**DOI:** 10.1556/168.2018.19.2.9

**Published:** 2018-12-30

**Authors:** F. Nieto-Rabiela, G. Suzán, A. Wiratsudakul, O. Rico-Chávez

**Affiliations:** 1 Departamento de Etología, Fauna Silvestre y Animales de Laboratorio, Facultad de Medicina Veterinaria y Zootecnia, Universidad Nacional Autónoma de México, Ciudad de México, México; 2 Department of Clinical Sciences and Public Health, Faculty of Veterinary Science, Mahidol University, Nakhon Pathom, Thailand

**Keywords:** Biogeographic scale, Disease ecology, Host environmental filtering, Niche theory, Zoogeographic scale

## Abstract

One of the main goals of community ecology is to measure the relative importance of environmental filters to understand patterns of species distribution at different temporal and spatial scales. Likewise, the identification of factors that shape symbiont metacommunity structures is important in disease ecology because resulting structures drive disease transmission. We tested the hypothesis that distributions of virus species and viral families from rodents and bats are defined by shared responses to host phylogeny and host functional characteristics, shaping the viral metacommunity structures at four spatial scales (Continental, Biogeographical, Zoogeographical, and Regional). The contribution of host phylogeny and host traits to the metacommunity of viruses at each spatial scale was calculated using a redundant analysis of canonical ordering (RDA). For rodents, at American Continental scale the coherence of viral species metacommunity increased while the spatial scale decreased and Quasi-Clementsian structures were observed. This pattern suggests a restricted distribution of viruses through their hosts, while in the Big Mass (Europe, Africa, and Asia), the coherence decreased as spatial scale decreased. Viral species metacommunities associated with bats was dominated by random structures along all spatial scales. We suggest that this random pattern is a result of the presence of viruses with high occupancy range such as rabies (73%) and coronavirus (27%), that disrupt such structures. At viral family scale, viral metacommunities associated with bats showed coherent structures, with the emergence of Quasi- Clementsian and Checkerboard structures. RDA analysis indicates that the assemblage of viral diversity associated with rodents and bats responds to phylogenetic and functional characteristics, which alternate between spatial scales. Several of these variations could be subject to the spatial scale, in spite of this, we could identify patterns at macro ecological scale. The application of metacommunity theory at symbiont scales is particularly useful for large-scale ecological analysis. Understanding the rules of host-virus association can be useful to take better decisions in epidemiological surveillance, control and even predictions of viral distribution and dissemination.

**Electronic Supplementary Material:**

Supplementary material is available for this article at 10.1556/168.2018.19.2.9 and is accessible for authorized users.

## Introduction

The distribution of species can either be explained by random processes, as neutral theory explains (Chave 2004, Hubbell 2005, Gonzalez 2009), or by environmental filters, as Niche Theory proposes (Leibold et al. 2004, Lorencio 2007, Morand and Krasnov 2010 chap. 7).

However, species distributions are influenced by the spatial and temporal contexts in which they occur, thus, neutral theory and niche theory should be tested across a different scale of analysis. There is evidence for the influence of different abiotic factors like temperature and elevation that can determine the organized distribution of species diversity, depending on the spatial scale studied (Rahbek and Graves 2001). The differences in species distributions between scales depend on three factors: physical barriers, the ability to co-occur, and their ability to disperse (Chase and Myers 2011). The diversity of symbionts has not been excluded from this type of analysis (Guernier et al. 2004). A large body of evidence suggest that the distribution of symbionts, defined as all organisms that must infect or inhabit hosts for at least part of their life cycle (Mihaljevic 2012), also responds to environmental filters determined by their hosts (Morand and Krasnov 2010, Mihaljevic 2012, Dallas and Presley 2014). Host phylogeny can act as an environmental filter to symbionts communites due to their interaction in the evolutionary history, co-adaptation and ecosystem process in the community (Streicker et al. 2010, Krasnov et al. 2014, Córdova-Tapia and Zambrano 2015). Similarly, the host functional characteristics can play a role as an environmental filter due to shared life histories or by spillover events (Davies and Pedersen 2008, Morand and Krasnov 2010).

We used viral communities associated with rodents and bats to analyse the effect of environmental filters. Rodents and bats are the most diverse orders of mammals thus they constitute suitable model taxa to explore the viral diversity providing relevant information to understand the dynamics of virus-host distribution (Lorencio 2007). These taxa have been recognized as the main reservoirs of a high number of zoonotic viruses, some of them with an enormous impact in public and animal health (Luis et al. 2013, 2015). Besides the individual approach, the study of the association between host and virus requires a clear understanding of the ecological context of infection and transmission be required (Woolhouse 2001, Suzán et al. 2015, Johnson et al. 2016). Because the host-virus system is intimately embedded within the communities, it is possible to recognize the existence of an organization in the distribution of viral diversity and later recognize the filters that allow or not to associate with a host (Suzán et al. 2015). The dispersion of a virus within a host community is accomplished through transmission events and may depend on the viral richness present in the community, so it is common to have multi-pathogen systems that can be considered as metacommunity (Suzán et al. 2015). Metacommunity theory implemented in viral communities at different spatial scales in combination with a redundancy analysis allows identifying the factors that facilitate virus distribution among hosts (Mihaljevic 2012, Dallas and Presley 2014, Suzán et al. 2015). Based on the metacommunity structures proposed by Leibold and Mikkelson (2002), and mechanisms for infection and prevalence proposed by Suzan et al. (2015), we can expect Random, Checkerboard and Clemenstian viral structures. A widespread distribution of abundant reservoir species are related with random structures while a limited viral distribution or a high viral specificity are related with Checkerboard or Clementsian structures (Suzán et al. 2015). The factors that shape viral communities and their distribution through their host at different spatial scales have not been studied. To measure the influence of the host phylogeny and functional characteristics of the host on viral community structure we hypothesized that both the expression of Clementsian structures based on the Niche Theory would prevail at different macroecological scales, and the host phylogeny will explain the viral metacommunity distribution as response of the shared host evolutionary histories and ecological relationships. We analyzed the contribution of phylogenetic and functional factors to the structure of viral metacommunities associated with rodents and bats. In our model, the viral community is defined as all viruses detected in each host species inside the geographic scale to analyze. In this analysis, the metacommunities are composed by viral communities linked by processes of dispersion and transmission between hosts.

To consider the spatial scale, the first analysis of viral metacommunities was performed on a Continental scale considering the ocean as the main geographic barrier for viral distribution. The subsequent macroecological scales were selected by their similarity on diversity composition as Biogeographical scales, recognized by their similarity in plant diversity (Cox 2001). Zoogeographical scales by their similarity in animal diversity (Holt et al. 2013), and Regional scales that contain local shared evolutionary histories (Holt et al. 2013).

## Methods

We constructed a database based on reports of viruses isolated or detected by molecular techniques in bats and rodents. We recorded the host species, the virus species, and viral family according to the International Committee of the Taxonomy of Viruses (ICTV) (https://talk.ictvonline.org/). The bat viruses information was collected from DbatVir database (http://www.mgc.ac.cn/DBatVir/), and data from rodents were collected by a literature search in Web of Science (https://webofknowledge.com), Elsevier (https://www.elsevier.com/advanced-search) and World Wide Science (https://worldwidescience.org/) with the keywords: “rodent”, “virus”, “PCR” and “wild”. The registered information was collected from years 1956 to 2015 and only studies with molecular techniques were considered. The geographic location was registered and classified into four spatial scales (Fig. 1): 1) According to the continental scale: America, Oceania and “Big Mass” that includes Europe, Africa, and Asia. 2) Biogeographical scale: Nearctic, Neotropical, Palearctic,

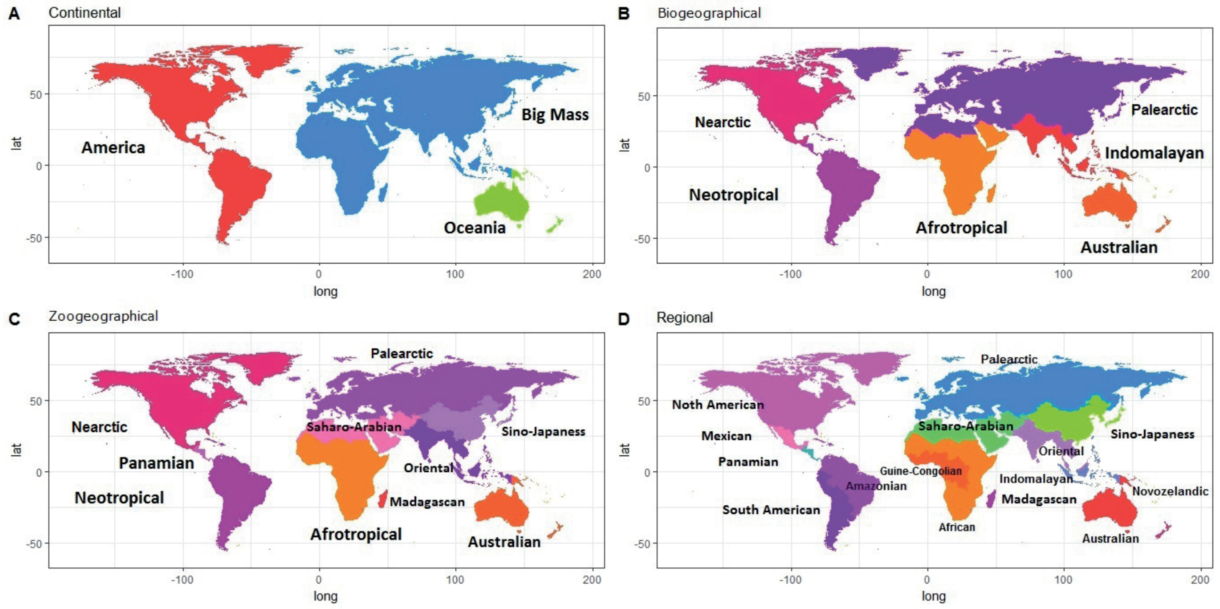

**Figure 1.** The framework of the spatial scales analyzed.

Afrotropical, Indomalayan and Australian (Cox 2001) 3) Zoogeographical scale: Nearctic, Panamian, Neotropical, Palearctic, Afrotropical, Oriental, Saharo-Arabian, Sino-Japaness, Madagascan and Australian (Holt et al. 2013). 4) Regional scales: North American, Mexican, Panamian, South American, Amazonian, Palearctic, African, Guine-Congolian, Madagascan, Indomalayan, Oriental, Saharo-Arabian, Sino-Japaness, Novozelandic, and Australian (Holt et al. 2013).

*Detection of metacommunity structures*

A presence-absence data matrix was constructed for each spatial scale and for each viral taxonomic level (species and family), where the virus was the column and the host the row. We obtained 62 rodent and 68 bat matrices, but due to the measures and the capacity of the algorithm performed in the metacommunity structure analysis, we could only obtain results of 24 and 38 matrices respectively. To evaluate the metacommunity structure of virus-host species form each matrix, we measure three properties. The coherence as the degree to which pattern can be collapsed into a single dimension, turnover counting the number of species replacements along the matrix and boundary cumpling measuring how the edges of species boundaries are distributed along this dimension (Leibold and Mikkelson 2002). The analysis of metacommunity structure was performed with the metacom package (Dallas 2014) implemented in R (R Core Team 2017), and the detection of metacommunity structure was using the Presley's decisions tree (Presley et al. 2010).

*Covariation of host characteristics with the viral metacommunity structure*

We measured the influence of the host phylogeny and functional characteristics of the host on viral community structure. The phylogenetic component was estimated taking the two first components of the PCoA analysis on the phylogenetic distance matrix, which was obtained by extracting the host species from the mammalian super-tree hosts (Bininda-Emonds et al. 2007) with the "picante" package. We include the body mass, litter size, number of litters per year and trophic guild, as host functional characteristics that may explain variation among hosts in viral community composition and their influence on the viral transmission. The variables were obtained from PanTHERIA database (Jones et al. 2009), Animal Diversity Web (http://animaldiversity.org) and Encyclopedia of Life (http://eol.org/). A redundant analysis of canonical ordination (RDA) has been performed to detect the relationship between the host phylogenetic component and host functional characteristics in the metacommunities from each spatial scale to obtain the explaining percentage on each one of detected metacommunity structures. The RDA was calculated with the algorithm varpart (Peres-Neto et al. 2006) of "vegan" package (Oksanen et al. 2016) implemented in R. This function performs a partition of the variation in community data for the explanatory variables.

## Results

*Database*

The rodents' database has 825 records, and includes 172 rodent host species, 124 virus species distributed in 23 viral families, of which 70 species and 14 families are zoonotic. The bats' database has 4,659 records and includes 220 host bats species, 174 virus species and 29 viral families of which 41 virus species and nine viral families are zoonotic.

*Metacommunity structures*

*Rodents.* We detected a Quasi-Clementsian structure in the metacommunities of viruses in the American Continent, and the distribution of these virus species was partly explained by host phylogeny (29%). The Big Mass scale showed a Clementsian structure explained by a low percentage of host functional characteristics (0.2%) (Table 1). Oceania continent was not included in the analysis because only four records were obtained. At Biogeographical level, a Clemenstian structure was detected in the Nearctic region, a Quasi-Clementsian structure in the Neotropic and Palearctic, and a random structure was found in the Afrotropical Region.

At Zoogeographical scale, only four regions were analyzed including Afrotropical with Quasi-Clemenstian structure, Nearctic with Clementsian and Palearctic and Oriental with Random structures. At Regional scale, three regions were analyzed including North Amercian, with Clementsian structure, African and Palearctic with Random structures. No relevant results were observed at metacommunities of viral families, where the random structures dominated in all scales except for Nearctic Biogeographic and Zoogeographic scale where a Quasi-Clementsian structure was detected.

*Chiropterans.* Except for the Big Mass scale where a Quasi-Clementsian structure was detected, the rest of the 19 viral species metacommunities analyzed showed Random structures. Oceania continent with 11 records was not possible to analyze. At biogeographic and zogeographical scale, Afrotropical and Palearctic regions presented a Quasi-Clemenstian structure, while we detected a Checkerboard structure in the Neotropical zoogeographical region. Only the Quasi-Clementsian structure of the Palearctic zone is maintained up to the Regional scale. At all scales, the host phylogeny and host functional characteristic explain the distribution of the viral families (Table 2).

## Discussion

*Rodents*

We observed different patterns at different scales of analysis. For example, in the American Continent the coherence increases as the geographic scale decreases, as shown in regional scales. Contrarily, in the Big Mass an opposite pattern was found; the coherence decrease with a decreasing geographic scale (Fig. 2). In the American continent these pat-

**Table 1.** Results of the analysis of coherence, range turnover, and boundary clumping for the viral metacommunities of rodents and results of RDA analysis. Abs, embedded absences; SD, standard deviation; df, degrees of freedom. Q-Clem.=Quasi-Clementsian.

| *Spatial Scale* / Community | Coherence | | | | Turnover | | | | Boundary clumping | | | Metacommunity | RDA Analysis | |
|---|---|---|---|---|---|---|---|---|---|---|---|---|---|---|
| | Abs | p | Mean | SD | Rep | p | Mean | SD | Index | p | df | Structure | varpart | % |
| *Continental* | | | | | | | | | | | | | | |
| America | 9 | 0.05 | 49.24 | 20.88 | | | | | | | | Random | phylo/ phylo+fun | 29 / 0.14 |
| Big Mass | 66 | 0.01 | 163.60 | 36.18 | 5884 | 0.03 | 10031.24 | 1874.44 | 2.18 | 0.00 | 41 | Clementsian | fun | 0.26 |
| *Biogeographical* | | | | | | | | | | | | | | |
| Nearctic | 17 | 0.01 | 68.61 | 20.94 | 1865 | 0.01 | 3656.22 | 711.63 | 3.41 | 0.00 | 39 | Clementsian | phylo/fun | 10 / 6.6 |
| Neotropic | 3 | 0.04 | 29.74 | 13.19 | 1329 | 0.13 | 1906.22 | 377.90 | 2.63 | 0.00 | 24 | Q-Clem. | phylo+fun | 0.9 |
| Afrotropical | 23 | 0.05 | 52.23 | 14.99 | | | | | | | | Random | 0 | |
| Palearctic | 96 | 0.00 | 256.43 | 36.10 | 6303 | 0.05 | 10560.87 | 2192.90 | 2.28 | 0.00 | 31 | Q-Clem. | phylo+fun | 0.9 |
| *Zoogeographical* | | | | | | | | | | | | | | |
| Palearctic | 136 | 0.36 | 156.01 | 21.69 | | | | | | | | Random | | |
| Afrotropical | 23 | 0.04 | 51.32 | 13.82 | 1242 | 0.46 | 1461.81 | 298.22 | 2.91 | 0.00 | 21 | Q-Clem. | phylo+fun | 1.6 |
| *Regional* | | | | | | | | | | | | | 0 | |
| North America | 17 | 0.01 | 66.50 | 19.94 | 1671 | 0.03 | 3192.51 | 684.30 | 3.72 | 0.00 | 37 | Clementsian | phylo/fun | 10.8 / 6.7 |
| African | 15 | 0.13 | 29.29 | 9.44 | | | | | | | | Random | phylo+fun | 19 |

**Table 2.** Results of the analysis of coherence, range turnover, and boundary clumping for the viral metacommunities of chiropters and results of RDA analysis. Abs, embedded absences; SD, standard deviation; df, degrees of freedom. Q-Clem.=Quasi-Clementsian.

| Spatial Scale / Community | Coherence | | | | Turnover | | | | Boundary clumping | | | Metacommunity | RDA Analysis | |
|---|---|---|---|---|---|---|---|---|---|---|---|---|---|---|
| | Abs | P | Mean | SD | Rep | P | Mean | SD | Index | P | df | Structure | varpart | % |
| *Continental* | | | | | | | | | | | | | | |
| America | 177 | 0.2187 | 130.817 | 37.5543 | | | | | | | | Random | fun | 0.8 |
| Big Mass | 782 | 0.32 | 866.77 | 85.77 | | | | | | | | Random | phylo+fun | 1.78 |
| *Biogeographical* | | | | | | | | | | | | | | |
| Nearctic | 32 | 0.55 | 41.88 | 16.39 | | | | | | | | Random | phylo+fun | 2.41 |
| Neotropic | 97 | 0.67 | 106.90 | 23.48 | | | | | | | | Random | phylo+fun | 1.7 |
| Afrotropical | 109 | 0.03 | 192.29 | 38.23 | 1606 | 0.14 | 4838.85 | 2167.68 | 7.77 | 0.00 | 14 | Q-Clem. | phylo+fun | 6.28 |
| Palearctic | 649 | 0.02 | 815.98 | 70.05 | 8618 | 0.05 | 27181.60 | 9668.56 | 8.15 | 0.00 | 23 | Q-Clem. | 0 | |
| Indomalayan | 28 | 0.23 | 37.93 | 8.29 | | | | | | | | Random | phylo+fun | 11.42 |
| *Zoogeographical* | | | | | | | | | | | | | | |
| Nearctic | 32 | 0.38 | 47.31 | 17.34 | | | | | | | | Random | phylo+fun | 2.02 |
| Neotropic | 111 | 0.00 | 52.91 | 18.90 | 413 | 0.13 | 1066.47 | 436.64 | 2.15 | 0.00 | 7 | Checkerboard | phylo+fun | 1.28 |
| Palearctic | 167 | 0.03 | 225.11 | 26.72 | 2150 | 0.19 | 3954.92 | 1368.93 | 3.95 | 0.00 | 17 | Q-Clem. | phylo+fun | 0.83 |
| Afrotropical | 88 | 0.02 | 153.69 | 27.66 | 768 | 0.09 | 3195.48 | 1421.86 | 7.03 | 0.00 | 14 | Q-Clem. | phylo+fun | 8.01 |
| Oriental | 28 | 0.25 | 37.81 | 8.48 | | | | | | | | Random | phylo+fun | 11.42 |
| Sino-Japanese | 322 | 0.20 | 368.74 | 36.43 | | | | | | | | Random | phylo / fun | 7.5 / 6.09 |
| *Regional* | | | | | | | | | | | | | | |
| South America | 12 | 0.25 | 27.11 | 13.20 | | | | | | | | Random | phylo+fun | 2.46 |
| African | 31 | 0.27 | 44.46 | 12.26 | | | | | | | | Random | phylo+fun | 1.79 |
| Guineo-Congolian | 27 | 0.06 | 48.07 | 11.22 | | | | | | | | Random | phylo/fun | 4.19/2.22 |
| Oriental | 11 | 0.48 | 14.03 | 4.33 | | | | | | | | Random | phylo+fun | 28.76 |

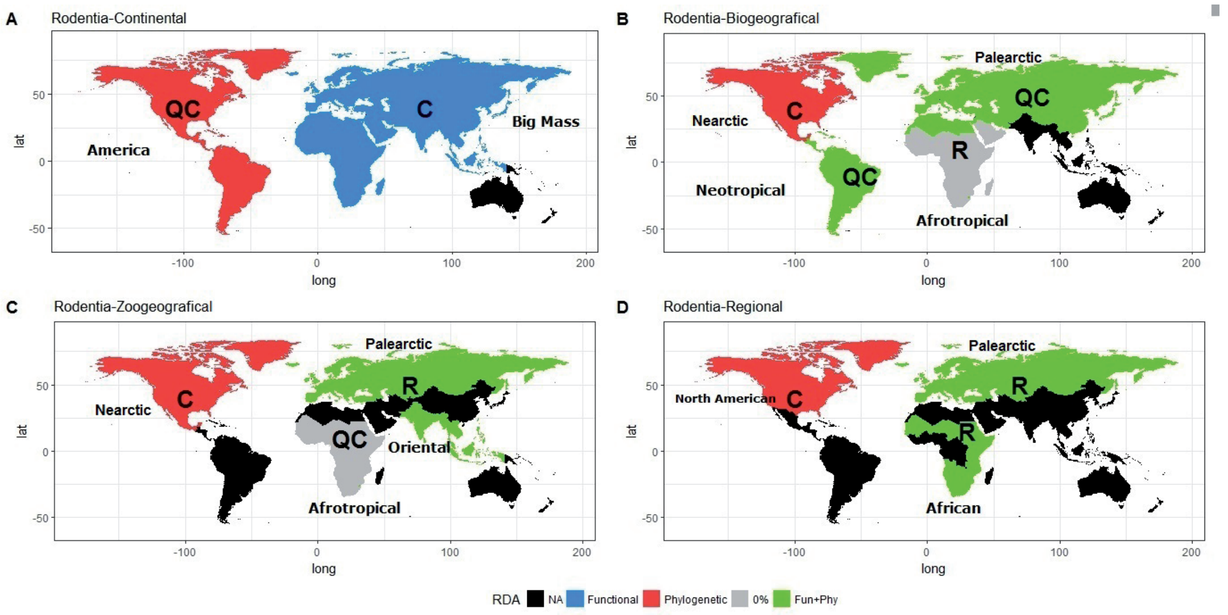

**Figure 2.** Structures of viral species metacommunities detected in rodents. Letters refer to the metacommunity structure: QC, Quasi-Clementsian; C, Clementsian, R, Random. The areas represent the variable that explains the viral distribution within the metacommunities obtained by the RDA analysis. Black – regions without enough data to detect a structure.

terns may be explaining by the high influence of the latitudinal gradient on the host distribution and by the island biogeography and the edge effect because the American Continent has a higher border surface (Lovejoy et al. 1986). Instead, the Big Mass surface allows a homogeneous host distribution (Buckley et al. 2010). At the Big Mass scale there are not strict physical barriers between bioregions and viruses are widely shared at the edges, mainly at the Palearctic-Afrotropical edge. These boundaries merged many years ago, forming a large area that allowed this exchange of viral diversity, so they are now arbitrary limits (Morand and Krasnov 2010). Therefore, it was possible to detect a Clementsian structure in the Big Mass metacommunity, but when the spatial scale decreased we observed a dominance of Random structures. At smaller communities, the characteristics of the host species are more widely shared without delimiting niches, and therefore the viral distribution depends on its capacity of dispersion rather than local filters.

In the Nearctic Biogeographic region, the Clementsian structure was explained by host phylogeny in 10% (Table 1), suggesting a phylogenetic signal and therefore, a higher specificity for host clades. The Quasi-Clementsian structure detected in the Neotropical Biogeographical region showed a weak response to environmental host filters (0.9%). When we compare these two regions, they show a response to a latitudinal diversity gradient (Kaufman 1995, Gaston 2000, Guernier et al. 2004), suggesting a greater diversity of host and viruses in the tropics probably influenced by a constant temperature (Morand and Krasnov 2010). This property facilitates the survival and viral mutations, facilitated by vectors proliferation, incrementing the chance of spillover, giving rise to generalist symbionts (Harvell et al. 2002).

The coherence in the structure of the Afrotropical zoogeographical region increases by the loss of the Middle East region, which was included at biogeographical scale and prevented the potential viral dispersion. Functional and phylogenetic characteristics explained the random structure detected in the African Region by 19% (Table 1). This percentage can be explained by the absence of *Thryonomys swinderianus* and *Xerus erythropus*, species in the African region who contained extreme values in their phylogenetic characteristics. It also shows a structure with a Clementsian tendency that is disturbed by *Mastomys natalensis*, a rodent associated with seven of the 14 viruses in this scale, in comparison with the remaining 21 rodents that host 1-4 virus species. Also, it is the only species in this level with reports of Banzi virus, Gairo virus, Mopeia and Morongo virus. Probably because of its anthropism and the most considerable sampling effort.

In Rodents we can assume that coherence decrease in viral families metacommunities may emerge due to the loss of information of viral species characteristics when viral families data was analyzed.

### *Chiropterans*

The metacommunity structure of viral species associated with bats was dominated by a Random structure, however, the distribution of the viruses is not aleatory because most of the metacommunities were explained by the host phylogeny and functional characteristics. Besides some viruses associated with Chiropterans are cosmopolitan, like Rabies virus, which have a wide range distribution within the metacommunitiy preventing the detection of a coherent structure.

In chiropterans, contrary to rodents, higher coherence at viral family scale suggests a clustering pattern of viral families. Besides, their classification of viral species is more specific, they even take the name of the host in which they were isolated.

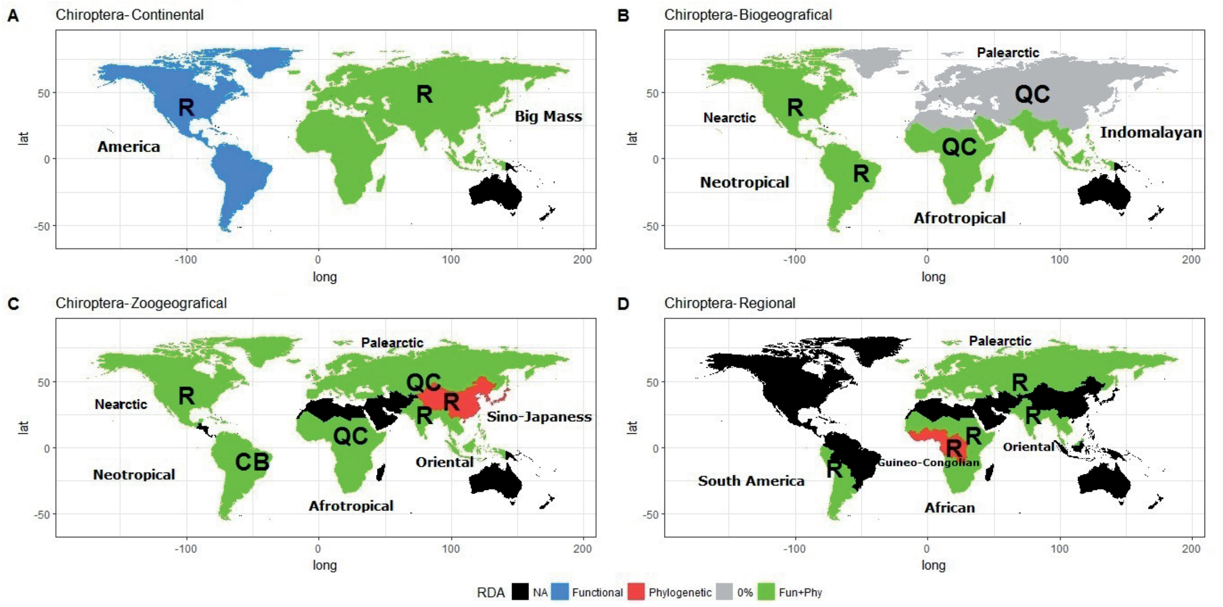

**Figure 3.** Structures of viral families metacommunities detected in chiropterans. Letters refer to the structure: QC, Quasi-Clementsian; C, Clementsian, R, Random, CB, Checkerboard. The colors represent the variable that explains the viral distribution within the metacommunities obtained by the RDA analysis. Black – regions without enough data to detect a structure.

The coherence of viral families metacommunities was only detectable at Biogeographical and Zoogeographical scales, while in the Continental and Regional scales the coherence cannot be observed. This result can be explained by removal of the host and viral families with a wide range of distribution, due that our model assumes a homogeneous distribution of host and viruses.

At America Continent scale, we detected several Random and Checkerboard structures in viral families metacommunities due to the presence of cosmopolitan viruses with a high sampling effort due to their public health relevance, such as the Rhabdoviridae and Coronaviridae families, found in 73% and 27% of the host species distributed in America respectively.

In the Palearctic and Afrotropical Biogeographic regions a Quasi-Clementsian structure was detected. When analyzing the Palearctic region at Zoogeographical scale, we can observe a coherence increase due to a separation of random structures which belong to the Zoogeographical regions Saharo-Arabica and Sino-Japanese (Fig. 3), with a low sampling effort. Despite this, the Sino-Japanese region is explained by phylogenetic (5.32%) and functional (6.09%) characteristics but separately, because the region contains extreme climates (Urteaga 1993) that could generate divergence of host and viruses (Gorman et al. 1992).

The random structure observed in Oriental Regional scale was explained by a high value (28.7%) of environmental host filter (phylogeny + functional characteristics). This result suggests that the absence of a coherent structure is resulted by the poor sampling effort in the area.

The virus families' distribution in the Quasi-Clementsian structure detected in the Afrotropical Biogeographic region is explained by environmental host filters (6.2%), however, when the geographic scale decreases the coherence increases with environmental host filter (8.2%). This increase of coherence can be explained by the absence of Madagascan region due to the geographic barrier that prohibits host migration. Meanwhile, the host migration between African and Guineo-Congolian Regions could be possible, explaining the decreases of coherence and the random structures detected in these two regions.

### *General patterns*

In general, a higher number of Clementsian and Quasi-Clementsian structures was observed in response to environmental host filters. The viral distribution responds primarily to dispersion filter by the geographic scale and secondly to the host characteristics, being affected by two types of simultaneous filters, at different spatial scales.

Random structures are explained by taking into account the information biases and the dynamism in which the ecosystems are involved, undergoing constant changes, of which we only manage to capture moments of their history. In spite of random spatial structures, host-virus relationships can still be highly specific, suggesting coevolution between hosts and viruses (Drexler et al. 2010) including coronaviruses (CoV, however our framework addressed to community scale cannot measure these events. Even so, the rules of community assembly are not a law, and they are only one of several mechanisms that alternate, so it proposes the predominance of Clements superorganism at macroecological level (Jaisson 2000). Thus, the study of such viral community assembly rules must be deepened to understand these processes.

Clarifying the influence of these environmental host filters, enables us to address the effect of differential study efforts and also to plan surveillance systems and responds to situations like emergent diseases. To understand and predict viral transmission dynamics it is important to identify those variables that explain viral distribution through their hosts. If the viral distribution is explained by the phylogenetic component we will be able to predict new hosts based on the phylogenetic similarity. Instead, if viral distribution is explained by the functional component we could predict new hosts based on functional traits similarity. We must not forget that multiple factors interact and affect the direction of changes in viral metacommunities so that their dynamism must be monitored and understood by multidisciplinary approaches.

## Conclusion

In general, it is more feasible to analyze the viral metacommunities associated with rodents at viral species scale by overlapping families, showing a weak phylogenetic signal between the host and the virus species. Bats, on the other hand, showed more order at the viral family level due to viral taxonomic classification, but also present more cosmopolitan viruses.

These inferences were based on the currently available data. Unfortunately, the present data set is insufficient to analyze the virus assemblages of small regions like the Australian and Madagascan due to the scarcity of data. Our data set is also likely to be biased because synanthropic species and viruses of public health relevance were sampled heavily, and this likely interferes with the influence of natural structures.

However, at the macroecological level, viral metacommunities associated with Rodentia and Chiropterans showed Clementsian structures or at least tended to them (quasi-Clementsians). The viral metacommunities mainly respond to spatial abiotic constraints, and secondarily to host environmental filters, which offer us an approach for the understanding of these clusters that explain a part of the set.

**Acknowledgments:** We are very grateful to PAPIIT (Project IA206416), Programa de Apoyo de los Estudios de Posgrado, UNAM, CONACYT, and Laboratorio de Ecología de Enfermedades y Una Salud, FMVZ, UNAM, especially to M. López Santana and D. Mendizabal Castillo for their contribution in databases construction.

## Appendix

**Supplementary Table 1.** Results of the analysis of coherence, range turnover, and boundary clumping for the viral families metacommunities of rodents and results of RDA analysis. Abs, embedded absences; SD, standard deviation; df, degree freedom.

**Supplementary Table 2.** Results of the analysis of coherence, range turnover, and boundary clumping for the viral species metacommunities of bats and results of RDA analysis. Abs, embedded absences; SD, standard deviation; df, degree freedom.

The file may be dowloaded from www.akademiai.com.

**Open Access.** This article is distributed under the terms of the Creative Commons Attribution 4.0 International License (https://creativecommons.org/licenses/by/4.0/), which permits unrestricted use, distribution, and reproduction in any medium, provided the original author and source are credited, you give a link to the Creative Commons License, and indicate if changes were made.

## Electronic supplementary material

Supplementary material, approximately 21 KB.

## References

[CR1] Bininda-Emonds ORP, Cardillo M, Jones KE, Macphee RDE, Beck RMD, Grenyer R, Price SA, Vos RA, Gittleman JL, Purvis A (2007). The delayed rise of present-day mammals. Nature.

[CR2] Buckley, L.B., T.J. Davies, D.D. Ackerly, N.J.B. Kraft, P. Susan, B.L. Anacker, H.V Cornell, E.I. Damschen, J. Grytnes, B.A. Hawkins, C.M. Mccain, P.R. Stephens and J.J. Wiens. 2010. Phylogeny, niche conservatism and the latitudinal diversity gradient in mammals. *Proc. Roy. Soc. Lond. B. Biol. Sci*. 277:rspb20100179. 10.1098/rspb.2010.0179 PMC2880153 20335205

[CR3] Chase JM, Myers JA (2011). Disentangling the importance of ecological niches from stochastic processes across scales. Phil. Trans. Royal Soc. B: Biol. Sci..

[CR4] Chave J (2004). Neutral theory and community ecology. Ecol. Lett..

[CR5] Córdova-Tapia F, Zambrano L (2015). La diversidad funcional en la ecología de comunidades. Revista Ecosistemas.

[CR6] Cox B (2001). The biogeographic regions reconsidered. J. Biogeogr..

[CR7] Dallas T (2014). Metacom: An R package for the analysis of metacommunity structure. Ecography.

[CR8] Dallas T, Presley SJ (2014). Relative importance of host environment, transmission potential and host phylogeny to the structure of parasite metacommunities. Oikos.

[CR9] Davies TJ, Pedersen AB (2008). Phylogeny and geography predict pathogen community similarity in wild primates and humans. Proc. Royal Soc. B: Biol. Sci..

[CR10] Drexler JF, Gloza-Rausch F, Glende J, Corman VM, Muth D, Goettsche M, Seebens A, Niedrig M, Pfefferle S, Yordanov S, Zhelyazkov L, Hermanns U, Vallo P, Lukashev A, Muller MA, Deng H, Herrler G, Drosten C (2010). Genomic characterization of severe acute respiratory syndrome-related coronavirus in European bats and classification of coronaviruses based on partial RNA-dependent RNA polymerase gene sequences. J. Virology.

[CR11] Gaston KJ (2000). Global patterns in biodiversity. Nature.

[CR12] Gonzalez, A. 2009. *Metacommunities: Spatial Community Ecology*. Encyclopedia of Life Sciences:1–8.

[CR13] Gorman OT, Bean WJ, Webster RG, Holland JJ (1992). Evolutionary processes in influenza viruses: divergence, rapid evolution, and stasis. Genetic Diversity of RNA Viruses.

[CR14] Guernier V, Hochberg ME, Guégan JF (2004). Ecology drives the worldwide distribution of human diseases. PLoS Biology.

[CR15] Harvell CD, Mitchell CE, Ward JR, Altizer S, Dobson AP, Ostfeld RS, Samuel MD (2002). Climate warming and disease risks for terrestrial and marine biota. Science.

[CR16] Holt BG, Lessard JP, Borregaard MK, Fritz SA, Araujo MB, Dimitrov D, Fabre PH, Graham CH, Graves GR, Jonsson KA, Nogues-Bravo D, Wang Z, Whittaker RJ, Fjeldsa J, Rahbek C (2013). Response to comment on “An Update of Wallace’s Zoogeographic Regions of the World.”. Science.

[CR17] Hubbell SP (2005). Neutral theory in community ecology and the hypothesis of functional equivalence. Funct. Ecol..

[CR18] Jaisson, P.C. 2000. La hormiga y el sociobiólogo. (No. 304.5 J3). México.

[CR19] Johnson PTJ, De Roode JC, Fenton A (2016). Why infectious disease research needs community ecology. Science.

[CR20] Jones KE, Bielby J, Cardillo M, Fritz S, O’Dell J, Orme CDL, Safi K, Sechrest W, Boakes EH, Carbone C, Connolly C, Cutts MJ, Foster JK, Grenyer R, Habib M, Plaster C, Price S, Rigby E, Rist J, Teacher A, Bininda-Emonds ORP, Gittleman JL, Mace GM, Purvis A (2009). PanTHERIA: a species-level database of life history, ecology, and geography of extant and recently extinct mammals. Ecology.

[CR21] Kaufman DM (1995). Diversity of new world mammals: universality of the latitudinal gradients of species and bauplans. J. Mammal..

[CR22] Krasnov BR, Pilosof S, Stanko M, Morand S, Korallo-Vinarskaya NP, Vinarski MV, Poulin R (2014). Co-occurrence and phylogenetic distance in communities of mammalian ectoparasites: Limiting similarity versus environmental filtering. Oikos.

[CR23] Leibold MA, Holyoak M, Mouquet N, Amarasekare P, Chase JM, Hoopes MF, Holt RD, Shurin JB, Law R, Tilman D, Loreau M, Gonzalez A (2004). The metacommunity concept: A framework for multi-scale community ecology. Ecol. Lett..

[CR24] Leibold MA, Mikkelson GM (2002). Coherence, species turnover, and boundary clumping: elements of meta-community structure. Oikos.

[CR25] Lorencio, C.G. 2007. Avances en ecología: hacia un mejor conocimiento de la naturaleza. Secretariado de Publicaciones de la Universidad de Sevilla.

[CR26] Lovejoy TE, Bierregaard RO, Rylands AB, Solé ME (1986). Edge and other effects of isolation on Amazon forest fragments. The Science of Scarcity and Diversity.

[CR27] Luis AD, Hayman DTS, O’Shea TJ, Cryan PM, Gilbert AT, Pulliam JRC, Mills JN, Timonin ME, Willis CKR, Cunningham AA, Fooks AR, Rupprecht CE, Wood JLN, Webb CT (2013). A comparison of bats and rodents as reservoirs of zoonotic viruses: are bats special? Proc. Royal Soc.B: Biol. Sci..

[CR28] Luis AD, O’Shea TJ, Hayman DTS, Wood JLN, Cunningham AA, Gilbert AT, Mills JN, Webb CT (2015). Network analysis of host-virus communities in bats and rodents reveals determinants of cross-species transmission. Ecol. Lett..

[CR29] Mihaljevic JR (2012). Linking metacommunity theory and symbiont evolutionary ecology. Trends Ecol. Evol..

[CR30] Morand S, Krasnov BR (2010). The Biogeography of Host–Parasite Interactions.

[CR31] Oksanen, A.J., F.G. Blanchet, M. Friendly, R. Kindt, P. Legendre, D. Mcglinn, P.R. Minchin, R.B.O. Hara, G.L. Simpson, P. Solymos, M.H.H. Stevens and E. Szoecs. 2016. Package “vegan” (Version 2.4-0). URL https://cran.r-project.org, https://github.com/vegandevs/vegan.

[CR32] Peres-Neto PR, Legendre P, Dray S, Borcard D (2006). Variation partitioning of species data matrices: estimation and comparison of fractions. Ecology.

[CR33] Presley SJ, Higgins CL, Willig MR (2010). A comprehensive framework for the evaluation of metacommunity structure. Oikos.

[CR34] R Core Team. (2017). R: A language and environment for statistical computing.

[CR35] Rahbek C, Graves GR (2001). Multiscale assessment of patterns of avian species richness. Proc. Nat. Acad. Sci..

[CR36] Streicker DG, Turmelle S, Vonhof MJ, Kuzmin IV, McCracken GF, Rupprecht CE (2010). Host phylogeny constrains cross-species emergence and establishment of rabies virus in bats. Science.

[CR37] Suzán G, García-Peña GE, Castro-Arellano I, Rico O, Rubio AV, Tolsá MJ, Roche B, Hosseini PR, Rizzoli A, Murray KA, Zambrana-Torrelio C, Vittecoq M, Bailly X, Aguirre AA, Daszak P, Prieur-Richard AH, Mills JN, Guégan JF (2015). Metacommunity and phylogenetic structure determine wildlife and zoonotic infectious disease patterns in time and space. Ecol. Evol..

[CR38] Urteaga, L. 1993. La Teoría De Los Climas Y Los Orígenes Del Ambientalismo. *Cuadernos criticos de geografia humana* XVIII:1–36.

[CR39] Woolhouse MEJ (2001). Population biology of multihost pathogens. Science.

