# Supplementary material for: Viral metacommunities associated to bats and rodents at different spatial scales
Source: Community Ecol. 2018 Dec 30;19(2):168–75. doi: 10.1556/168.2018.19.2.9 (PMC7091747; doi:10.1556/168.2018.19.2.9)
Supplement: Supplementary file 1 — Supplementary material, approximately 21 KB. [file 42974_2018_19020168_MOESM1_ESM.pdf]

Supplementary material

Supplemental Table 1. Results of the analysis of coherence, range turnover, and boundary clumping for the viral families metacommunities of rodents and results of RDA analysis. Abs, embedded absences; SD, standard deviation; df, degree freedom.

***Spatial Scale* / Community**	**Coherence**				**Turnover**				**Boundaring**			**Metacommunity Structure**	**RDA Analysis**	
	**Abs**	**p**	**Mean**	**SD**	**Rep**	**p**	**Mean**	**SD**	**Index**	**p**	**df**		**varpart**	**%**
Continental														
America	97	0.746	87.94	28.00								Random	phylo / phylo+fun	6.43 / 1.84
Big Mass	124	0.536	141.39	28.08								Random	phylo+fun / phylo	1.53 / 0.32
Biogeographic														
Neartic	39	0.020	82.90	18.82	1959	0.18	3016.19	795.09	2.73	0.00	14	Quasi-Clementsian	phylo / fun	13.12 / 7.31
Neotropic	18	0.733	22.16	12.19								Random	phylo+fun	3.02
Paleartic	170	0.792	177.11	26.94								Random	phylo+fun	0.94
Zoogeographic														
Neartic	39	0.025	84.11	20.07	1959	0.21	2980.01	819.54	2.73	0.00	14	Quasi-Clementsian	phylo / fun	13.12 / 7.31
Afrotropical	27	0.604	332.11	9.85								Random	0	
Paleartic	118	0.111	94.11	14.98								Random	phylo+fun	4
Sino-Japanese	18	0.450	13.46	6.01								Random	fun	4

Supplemental Table 2. Results of the analysis of coherence, range turnover, and boundary clumping for the viral species metacommunities of bats and results of RDA analysis. Abs, embedded absences; SD, standard deviation; df, degree freedom.

***Spatial Scale* / Community**	**Coherence**				**Turnover**				**Boundaring**			**Metacommunity**	**RDA Analysis**	
	**Abs**	**p**	**Mean**	**SD**	**Rep**	**p**	**Mean**	**SD**	**Index**	**p**	**df**	**Structure**	**varpart**	**%**
Continental														
America	179	0.63	200.99	45.35								Random	fun	0.88
Big Mass	1699	0.02	2107.66	172.21	188189	0.08	278104.12	52119.79	12.51	0.00	79	Quasi-Clementsian	phylo+fun	0.83
Biogeographic														
Neartic	33	0.20	58.08	19.49								Random	phylo+fun	2.39
Neotropic	102	0.12	148.56	29.79								Random	phylo+fun	1.71
Paleartic	2253	0.90	2220.65	267.49								Random	phylo / phylo+fun	1.73 / 0.69
Afrotropical	354	0.86	345.29	48.35								Random	phylo+fun	6.12
Indomalayan	32	0.12	55.74	15.29								Random	phylo+fun	8.96
Zoogeographic														
Neartic	52	0.49	66.98	21.50								Random	phylo+fun	2
Neotropic	50	0.69	57.90	19.78								Random	phylo+fun	1.28
Paleartic	398	0.05	516.11	60.84								Random	phylo+fun	0.65
Afrotropical	289	0.66	270.42	41.61								Random	phylo+fun	7.41
Oriental	32	0.11	56.85	15.53								Random	phylo+fun	8.96
Sino-Japanese	719	0.46	792.10	99.58								Random	fun / phylo	6.09 / 5.32
Regional														
North America	12	0.27	28.80	15.24								Random	phylo+fun	9
South America	14	0.31	28.43	14.20								Random	0	
African	74	0.16	100.39	18.96								Random	phylo+fun	9.62
Guineo-Congolian	93	0.07	66.38	14.45								Random	phylo+fun	19
